# In/Ga-Doped Si as Anodes for Si–Air Batteries with Restrained Self-Corrosion and Surface Passivation: A First-Principles Study

**DOI:** 10.3390/molecules28093784

**Published:** 2023-04-27

**Authors:** Dongxu Wang, Tingyu Zhao, Yingjian Yu

**Affiliations:** College of Physics Science and Technology, Kunming University, Kunming 650214, China

**Keywords:** Si–air batteries, adsorption energy, band gap, density of states, I-V curves

## Abstract

Silicon–air batteries (SABs) are attracting considerable attention owing to their high theoretical energy density and superior security. In this study, In and Ga were doped into Si electrodes to optimize the capability of Si-air batteries. Varieties of Si-In/SiO_2_ and Si-Ga/SiO_2_ atomic interfaces were built, and their properties were analyzed using density functional theory (DFT). The adsorption energies of the SiO_2_ passivation layer on In- and Ga-doped silicon electrodes were higher than those on pure Si electrodes. Mulliken population analysis revealed a change in the average number of charge transfers of oxygen atoms at the interface. Furthermore, the local device density of states (LDDOS) of the modified electrodes showed high strength in the interfacial region. Additionally, In and Ga as dopants introduced new energy levels in the Si/SiO_2_ interface according to the projected local density of states (PLDOS), thus reducing the band gap of the SiO_2_. Moreover, the I-V curves revealed that doping In and Ga into Si electrodes enhanced the current flow of interface devices. These findings provide a mechanistic explanation for improving the practical efficiency of silicon–air batteries through anode doping and provide insight into the design of Si-based anodes in air batteries.

## 1. Introduction

Due to the rapid economic development over the years, the excessive use of nonrenewable energy sources, such as oil and coal, has led to environmental problems due to the excessive emission of gases, such as CO_2_. The development and use of clean energy can effectively alleviate the global problem of CO_2_ emissions [1,2]. Lithium-ion batteries (LIBs) have been widely used in mobile devices and electric vehicles owing to their advantages, such as portability and long lifetime [3,4,5,6,7]. Generally, conventional lithium-ion battery technology is limited by its performance (energy density of 350 Wh/kg) [8]. In addition, a shortage of resources and rising costs have limited the further application of LIBs in large-scale storage systems. Among next-generation batteries, metal–air batteries (MABs) can directly use atmospheric oxygen without storing the cathode reactant inside the batteries, thus endowing them with the advantage of high energy density [9]. Li, Zn, Al, and other elements are often used as anode materials for MABs, and their theoretical energy densities are within the range of 6001~9677 Wh/L. However, metal anodes of MABs are associated with several challenges, such as dendrite formation on Li and Zn, which may cause safety problems in MABs [8,9].

In addition to metal anodes, silicon also has application potential in air batteries. Si–air batteries were first invented by Ein-Eli et al. in 2009 [10]. To date, researchers have continued to study the working mechanism of these batteries and obtained average operating potentials of approximately 1.1 V with long-term cycles [11]. Silicon–air batteries have a high theoretical energy density of 8461 Wh/kg. Because Si is one of the most abundant elements on the Earth, its use as an anode material will somewhat reduce the cost and alleviate the resource shortage of other anode materials [8]. Alkaline Si–air battery mainly comprises three basic parts: a silicon anode, a potassium hydroxide solution electrolyte, and an air cathode. During the discharge process, the passivation reaction at the anode interface forms a dense oxide layer on the anode, thereby insulating discharge reactants. The insulated reactants can inhibit further discharge and even lead to the failure of batteries. In addition, the self-corrosion (hydrogen evolution reaction) between the silicon anode and water molecules in the electrolyte cannot be neglected. The corrosion reaction of silicon electrodes in an alkaline solution damages the anode material and reduces the specific capacity of the batteries [12,13,14]. The reaction equations in Si–air batteries are expressed as follows:

Anode: Si + 4OH^−^ ⇋ Si(OH)_4_ + 4e^−^ (1)

Cathode: O_2_ + 2H_2_O + 4e^−^ ⇋ 4OH^−^ (2)

Passivation: Si(OH)_4_ → SiO_2_ + 2H_2_O (3)

Silicate formation: Si(OH)_4_ + 2OH^−^ ⇋ SiO_2_(OH)_2_^2−^ + 2H_2_O (4)

Self-corrosion: Si + 2OH^−^ +2H_2_O ⇋ SiO_2_(OH)_2_^2−^ + 2H_2_ (5)

Because the half-cell potentials of the cathode and anode are 0.4 V and −1.69 V, respectively, the voltage of the alkaline silicon–air cell can theoretically reach 2.09 V, and the open-circuit voltage can be approximately 1.5 V [13,14]. The discharge voltage of 1.1 V mentioned in previous works is lower than the theoretical value. In aqueous Si–air batteries, the passivation of the anode interface and the self-corrosion loss of the silicon electrode are inevitable. A higher potential difference and an insulating oxide layer inhibit the electron transfer at the interface, and the self-discharge of the silicon anode reduces the efficiency of the anode. The passivation and self-corrosion are closely related to operating voltages that are lower than the theoretical values. Our work focuses on the mitigation of the passivation and self-corrosion of silicon–air batteries in an alkaline electrolyte environment.

For the protection of electrodes, many researchers have started with doping and surface coating to improve the performance of electrodes and increase the actual energy density of various batteries [15,16,17,18,19,20,21]. Because silicon is an intrinsic semiconductor, its electrical conductivity is lower than that of metals. To address this problem, researchers have modified silicon anode through anode doping modification to increase the electron transfer rate, thus enhancing the interfacial reaction. Doping modification is achieved by influencing the energy band of semiconductor materials by adding other elements to change their Fermi energy level. Eichel et al. investigated the effect of anode doping by using As and B on non-aqueous silicon–air batteries. They found that As-doped silicon anodes had a higher discharge potential than B-doped silicon anodes. Moreover, the silicon anode doped with high B content significantly suppressed anode self-corrosion, while the As-doped silicon anode was more prone to corrosion at open-circuit voltage (OCV). The longer the OCV is maintained, the higher the corrosion rate [22]. Eichel et al. also studied the effect of different doping types of Si anodes on the performance efficiency and corrosion of silicon–air batteries. They found that As-doped silicon anodes had the highest OCV and discharge potentials in silicon–air batteries, and it was almost independent of the silicon crystal orientation [23]. The above studies on the discharge behavior and performance of doping modification of anodes of SABs have provided insight into improving the anode performance and the energy density of SABs. However, there is an insufficient theoretical explanation at the atomic level for the working mechanism of doped silicon-based composite anodes. Si modified with different dopants has been modeled in a few previous works. Single-atom doping of Zn and Fe has been performed to suppress interfacial passivation. In addition, silicon has been replaced with different quantities of Ge atoms to regulate the interface passivation and electron transfer [24,25]. The above two works focus on the suppression of interface passivation through doping; however, there is still a lack of theoretical research on another important issue, e.g., corrosion, in silicon–air batteries. In and Ga, as metal elements of the third main group, are often considered doping alternatives for electrode modification owing to their excellent properties, such as low toxicity, good ductility, high plasticity, and satisfactory stability in an alkaline environment [26,27,28,29,30]. Some experimental methods for In and Ga, such as chemical vapor deposition, low-temperature solution synthesis, and spraying, have been developed and implemented to design new composites [28,30,31,32]. Alshareef et al. designed a novel indium-coated carbon–zinc composite anode with high kinetics to further improve the cycling stability of energy-based zinc ion batteries and power-based zinc ion capacitors [28]. Gao et al. prepared an anode with Ga-In-Sn-Zn solid–liquid composite (SLC) material for a zinc ion battery, which endowed the new composite electrode with a good adsorption ability of zinc ions and a relatively low metal-ion migration energy barrier [30].

This work aimed to modify silicon anodes through doping modification to effectively inhibit anode passivation and self-corrosion during battery discharging. In this study, we chose In and Ga metals as the dopants with low storage in the Earth’s crust. The use of the dopants may increase the fabrication cost of composite silicon anodes to some extent. As a result, a small amount of dopant is needed to enhance the anode and battery performances, thus reducing the cost of the dopants and battery.

In this work, silicon models were doped using In and Ga. Si–In/SiO_2_ and Si–Ga/SiO_2_ interface models were developed and further studied using the DFT calculation. By comparing the adsorption energy of the interfacial oxide layer on In- and Ga-doped Si anode, we inferred that In and Ga as dopants would efficaciously inhibit the passivation on the anode. Moreover, the dopants significantly affected the electron density, density of states, and band gap width close to the Si/SiO_2_ interface and restrained the adsorption of the H_2_O molecule, thus inhibiting self-corrosion. Finally, the I-V profiles showed that the dopants would enhance the electron-transferring kinetics across the anode interface. This work provides an anode modification strategy to improve the performance of SABs and broaden the application of In and Ga in air batteries.

## 2. Results and Discussion

**Figure 1:**
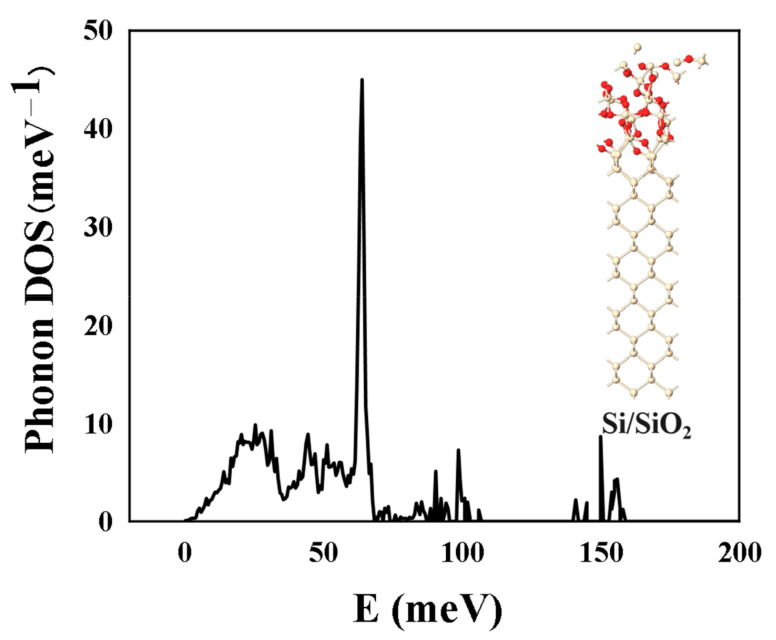
Phonon DOS of Si/SiO_2_ model after structural optimization.

We calculated the phonon density of states (PDOS) for the Si/SiO_2_ model (Figure 1). In the negative frequency region, the PDOS was zero, i.e., the calculation result had no negative frequency, indicating that the system was in the lowest energy state, which confirmed that the system was stable enough from a certain perspective.

**Figure 2:**
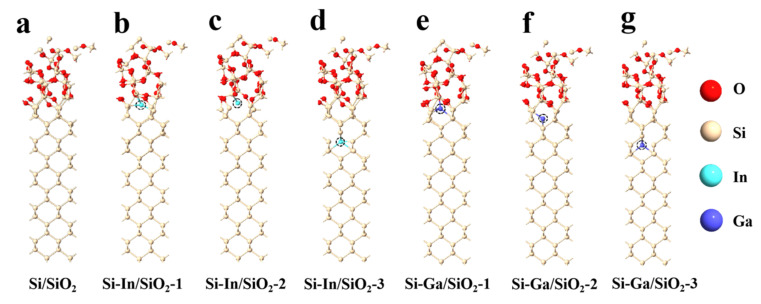
Atomic configurations of (**a**) Si/SiO_2_, (**b**) Si–In/SiO_2_-1, (**c**) Si–In/SiO_2_-2, (**d**) Si–In/SiO_2_-3, (**e**) Si–Ga/SiO_2_-1, (**f**) Si–Ga/SiO_2_-2, and (**g**) Si–Ga/SiO_2_-3 interfaces. Red, yellow, cyan, and blue balls represent O, Si, In, and Ga atoms, respectively.

**Figure 3:**
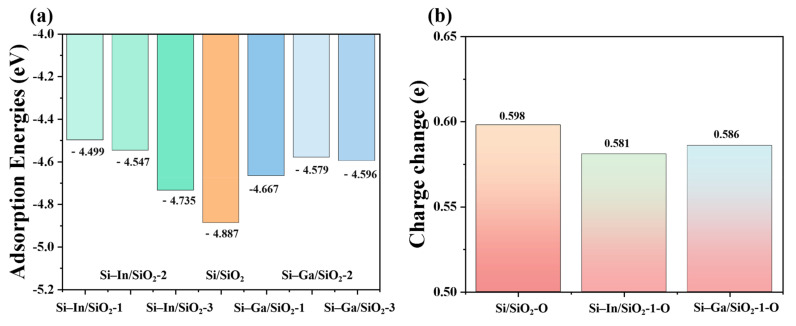
(**a**) Adsorption energies of SiO_2_ layer on pure Si and doped Si electrode. (**b**) Mulliken population analysis of the average charge change in O atoms at the interface of the Si/SiO_2_, Si–In/SiO_2_-1, and Si–Ga/SiO_2_-1 models.

The configurations of the Si/SiO_2_ atomic interface doped using In and Ga at different positions are displayed in Figure 2. The Si/SiO_2_ interface in Figure 2a shows that the Si atoms at the electrode surface are bonded to Si and O atoms in the oxide layer. Then, one In atom was doped at three different positions close to the interface, as exhibited in Figure 2b–d. According to Si–In/SiO_2_-1 model, there was one In–Si bond with a bond length of 2.75 Å between the In and the silicon atoms in the silicon anode, and three In-O bonds with bond lengths of 2.12 Å, 2.73 Å, and 2.59 Å. In the case of Si–In/SiO_2_-2, the In–Si bond with a length of 2.59 Å was between In and the silicon atoms on the passivation layer at the interface. According to the Si-In/SiO_2_-3 model, there were six In–Si bonds with bond lengths of around 2.67 Å. Similarly, one Ga atom was decorated at different positions on the Si substrate, as shown in Figure 2e–g. In terms of the Si–Ga/SiO_2_-1 interface, the length of the Ga–O bond was 1.99 Å. Meanwhile, Ga atoms were bonded to three silicon atoms in the silicon anode, and the three Ga–Si bond lengths were 2.47 Å, 2.55 Å, and 2.49 Å, respectively. The Ga atom in Si–Ga/SiO_2_-2 combined with two silicon atoms in the silicon anode, and the bond lengths of the two Ga–Si bonds were 2.58 and 1.99 Å. Finally, in the Si–Ga/SiO_2_-3 model, the average bond length of the four Ga–Si bonds was 2.52 Å. In order to quantitatively analyze the combination between different silicon substrates and silicon dioxide layers, the adsorption energy between them was computed using the following equation:

(6) $${\mathrm{Ea}:}_{\mathrm{Si}/{\mathrm{SiO}}_{2}}=(E_{\mathrm{Si}/{\mathrm{SiO}}_{2}}\text{-}E_{\mathrm{Si}}\text{-}E_{{\mathrm{SiO}}_{2}})/n$$

(7) $${\mathrm{Ea}:}_{\mathrm{Si}\text{-}M/{\mathrm{SiO}}_{2}}=(E_{\mathrm{Si}\text{-}M/{\mathrm{SiO}}_{2}}\text{-}E_{\mathrm{Si}\text{-}M}\text{-}E_{{\mathrm{SiO}}_{2}})/n$$

where $E_{\mathrm{Si}/{\mathrm{SiO}}_{2}}$ and $E_{\mathrm{Si}\text{-}M/{\mathrm{SiO}}_{2}}$ represent energies of Si/SiO_2_, Si–In/SiO_2_ and Si–Ga/SiO_2_ models, $E_{\mathrm{Si}}$ and $E_{\mathrm{Si}\text{-}M}$ represent the energy of pure Si substrate and the Si substrate doped by In or Ga, $E_{{\mathrm{SiO}}_{2}}$ is the energy of the oxide part, and n represent the number of oxide units (n is equal to 16 here), respectively [32]. The adsorption energies of all models are shown in Figure 3a. The numerical value of the Si/SiO_2_ configuration was −4.887 eV, which was the lowest among all the interface models, indicating that the SiO_2_ passivation layer was significantly inclined to accumulate the pure Si electrode, thus limiting the discharge time of the Si–air battery. After In atom was doped at three different positions, the adsorption energies of the three models were increased to −4.499 eV, −4.547 eV, and −4.735 eV, respectively. The results showed that the doping could weaken the combination of the Si anode and passivation layer, thus somewhat surface passivation. The closer the doping position of the In atom is to the interface, the more significant the anti-passivation effect. The adsorption energies of Ga-doped interface models were also calculated, which revealed that Ga dopants would also suppress surface passivation. A diminished silica layer promoted electron transfer kinetics and improved the performance of SABs. In addition, Mulliken population analysis on the change in average charge of the interfacial oxygen atoms is displayed in Figure 3b. The average charge change in Si/SiO_2_ atomic model was 0.598, which is higher than those in In/Ga-doped interface models (0.581 and 0.586), indicating that the weaker binding between the SiO_2_ layer and in Ga-doped Si electrode was weak.

**Figure 4:**
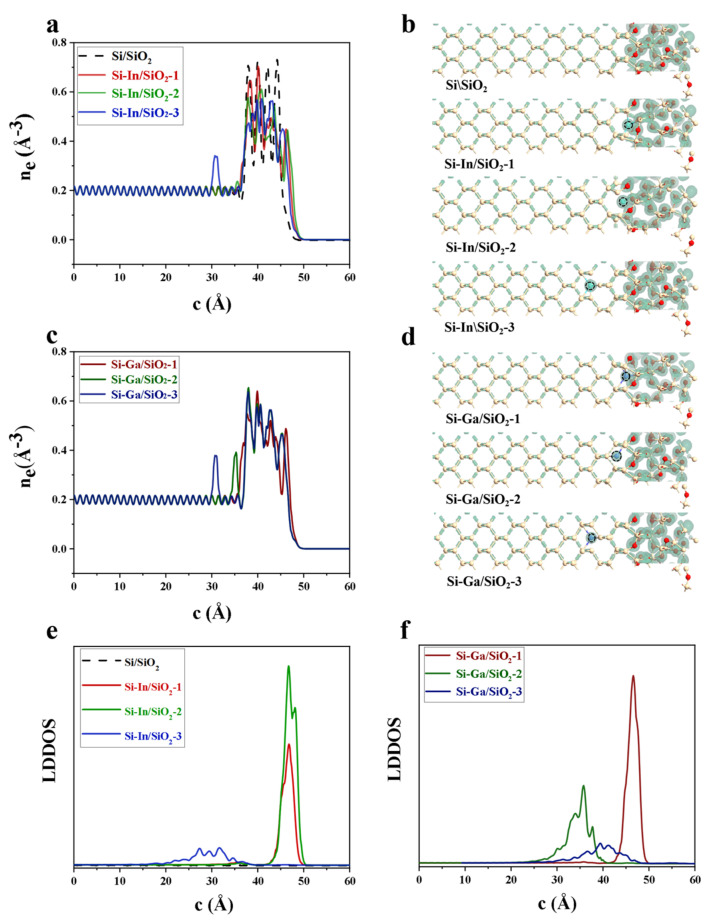
One-dimensional average projection of the electron density of (**a**) the Si/SiO_2_, Si–In/SiO_2_-1, Si–In/SiO_2_-2, and Si–In/SiO_2_-3 models along the c-axis and (**c**) the Si–Ga/SiO_2_-1, Si–Ga/SiO_2_-2, and Si–Ga/SiO_2_-3 models. Isosurface of the electron density with an isovalue of 0.5 Å^−3^ in (**b**) the Si/SiO_2_, Si–In/SiO_2_-1, Si–In/SiO_2_-2, and Si–In/SiO_2_-3 models and (**d**) the Si–Ga/SiO_2_-1, Si–Ga/SiO_2_-2, and Si–Ga/SiO_2_-3 models. The LDDOS of (**e**) the Si/SiO_2_, Si–In/SiO_2_-1, Si–In/SiO_2_-2, and Si–In/SiO_2_-3 models and (**f**) the Si–Ga/SiO_2_-1, Si–Ga/SiO_2_-2, and Si–Ga/SiO_2_-3 models.

The effects of In and Ga as doping species on the electron density and local device density of states (LDDOS) of various interface models are shown in Figure 4. According to the electron densities averagely projected along the c-axis and isosurfaces of the electron density in Figure 4a–d, it can be seen that the electron density close to In and Ga dopants is significantly higher than that in the Si electrode for Si–In/SiO_2_-3, Si–Ga/SiO_2_-2 and Si–Ga/SiO_2_-3 models. As shown in Figure 4e,f, the modified interface models exhibited a higher LDDOS peak at the doping location than the Si/SiO_2_ interface, indicating that more density of states were introduced by In and Ga dopants.

**Figure 5:**
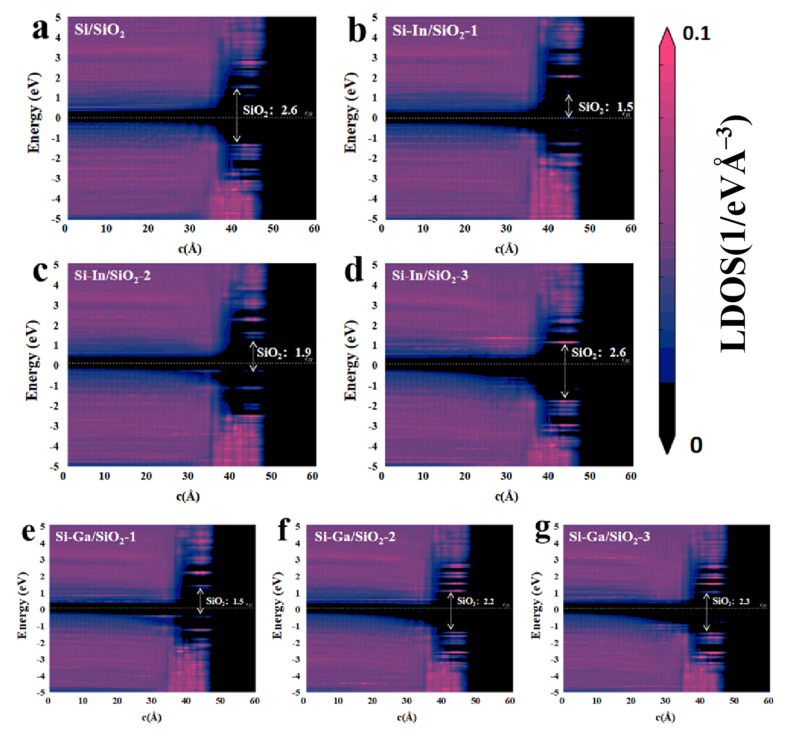
The PLDOS of the (**a**) Si/SiO_2_, (**b**) Si–In/SiO_2_-1, (**c**) Si–In/SiO_2_-2, (**d**) Si–In/SiO_2_-3, (**e**) Si–Ga/SiO_2_-1, (**f**) Si–Ga/SiO_2_-2, and (**g**) Si–Ga/SiO_2_-3 models. The dashed arrow represents the SiO_2_ forbidden band width.

To clarify the effect of doping In and Ga on the interfacial bandgap, the PLDOS of various models was calculated, as shown in Figure 5. The calculation results revealed the band gap of Si substrates and oxide layers. As shown in Figure 5a, the Si/SiO_2_ model displayed a band gap width of 2.6 eV for SiO_2_. After the electrode was doped with an In atom, the band gap decreased to 1.5 eV or 1.9 eV, with some introduced energy levels for the first two Si–In/SiO_2_ interfaces, as visualized in Figure 5b,c. The In dopant in the third model was relatively far from the interface. Therefore, the band gap width was similar to that of the Si/SiO_2_ model in the SiO_2_ region. Similar phenomena can be observed in–Ga-doped models in Figure 5e–g, yielding a bandgap width between 1.5 and 2.3 eV. Thus, the influence of doping on the forbidden band width of SiO_2_ decreased as the doping position moves moved from the interface. In this study, only the GGA scheme was used to calculate the band gap without correction. Generally, the band gaps calculated using GGA are expected to be lower than the experimental results. Although differences existed between the band gap obtained using GGA and the experimental results, a qualitative analysis could also be used to draw a credible conclusion [33]. The band gap calculation in this paper will guide the further experiment. In general, In and Ga dopants somewhat decreased the band gap width at the interface decrease to a certain extent, thus improving electron transfer and favoring the performance of SABs.

**Figure 6:**
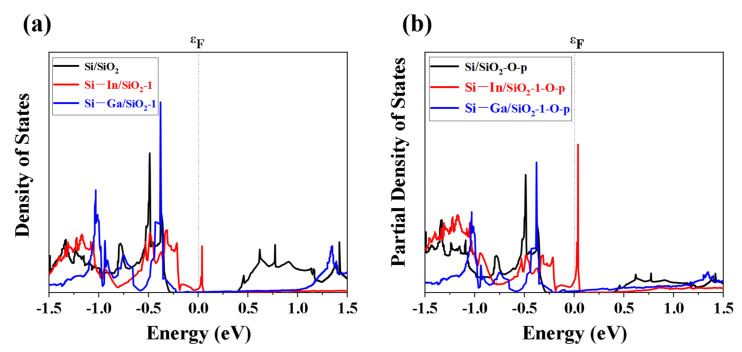
(**a**) DOS of the SiO_2_ layer and (**b**) partial DOS of p-orbital of O atoms in the Si/SiO_2_, Si–In/SiO_2_-1, and Si–Ga/SiO_2_-1 models.

In addition, we calculated and analyzed the density of states (DOS) of silica and p-orbital of oxygen atoms in silica to illustrate the mechanism of the effect of doping In and Ga atoms on the band gap of oxide layers. The similarity in DOS profiles indicates that the partial density of states of the O-p orbital dominates the band gap and DOS of the oxide layer, as shown in Figure 6a,b. It is worth noticing that the doping of In and Ga atoms significantly affected the band gap and the DOS of the model. The peak at ~ 0 eV corresponded to the position where the new energy level appeared in the first In-doped interface in Figure 5b. Furthermore, the peak at ~ −0.4 eV corresponded to the new energy level in the first Ga-doped interface in Figure 5e.

**Figure 7:**
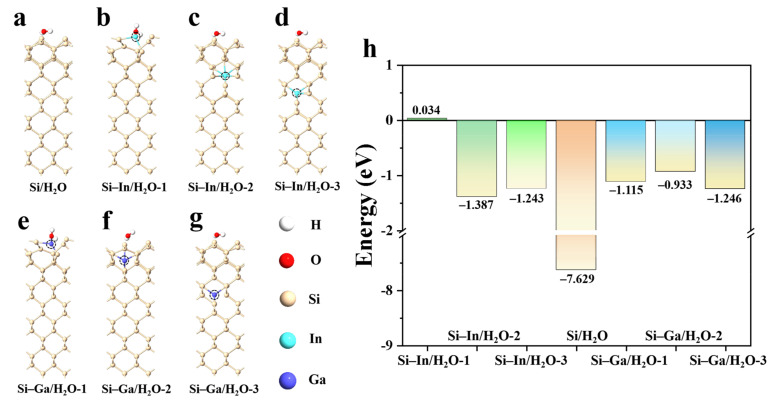
Atomic configurations of (**a**) Si/H_2_O, (**b**) Si–In/H_2_O-1, (**c**) Si–In/H_2_O-2, (**d**) Si–In/H_2_O-3, (**e**) Si–Ga/H_2_O-1, (**f**) Si–Ga/H_2_O-2, and (**g**) Si–Ga/SiO_2_-3 interfaces. White, red, yellow, brown, cyan, and blue balls represent H, O, Si, In, and Ga atoms, respectively. (**h**) Adsorption energies of the H_2_O molecule on pure Si and various doped Si electrode.

To investigate the effect of doping on the inhibition of self-corrosion, as water plays a vital role in the self-corrosion reaction, we used one water molecule to simulate the water-contacting surface of the silicon electrode under aqueous conditions. Then, we calculated the adsorption energy of the water molecule at the interface of different models for further analysis. A water molecule can somewhat reflect the water molecules in the electrolyte. Pure Si and doped Si electrode models containing an H_2_O molecule were constructed, as visualized in Figure 7a–g. The adsorption energies of one H_2_O molecule on various Si models are shown in Figure 7h. The result for the pure silicon electrode was −7.629 eV, confirming the strong binding between the H_2_O molecule and Si electrode. After the Si electrode was doped, the adsorption energy of water molecules at the interface of the Si–In/H_2_O-1 model became the highest (0.034 eV), which indicates that the repulsion against the In atoms doped at other locations also contributed to the increase in the adsorption energy of water molecules. The adsorption energies of water molecules in the Si–In/H_2_O-2 and Si–In/H_2_O-3 models were −1.387 eV and −1.243 eV, respectively. A similar situation was observed when the Ga atom was doped, and the adsorption energy of water molecules in Si–Ga/H_2_O-1, Si–Ga/H_2_O-2, and Si–Ga/H_2_O-3 models were −1.115 eV, −0.933 eV, and −1.246 eV, respectively, indicating that the Ga dopant would also contribute to the increase in the adsorption energy of water molecules. Given the result of the interfacial adsorption energy of water molecules in the Si/H_2_O model, the doped Si electrode had a stronger repulsive force on the water molecules, thus effectively inhibiting the self-corrosion reaction of the electrode in the alkaline electrolyte. Water is an important participant in the self-corrosion effect. Doping can inhibit the binding of water molecules to the interface to reduce side reactions and loss of anode material, thus enhancing the energy density of SABs.

**Figure 8:**
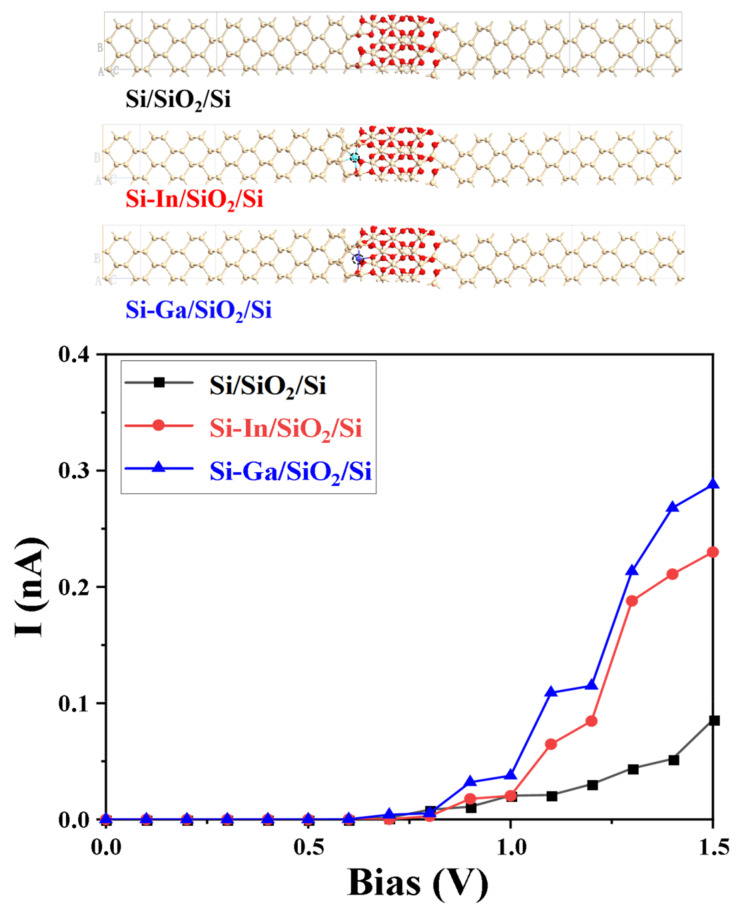
Atomic configurations and I-V curves of the Si/SiO_2_/Si, Si–In/SiO_2_/Si, and Si–Ga/SiO_2_/Si devices.

Finally, from the perspective of a comparative study of the nature of electron transfer, I-V curves were also analyzed by constructing pure and In/Ga-doped Si/SiO_2_/Si devices, as exhibited in Figure 8. By considering the actual potential of SABs into account, the voltage bias was set to 0–1.5 V. We observed that currents were close to zero when the bias voltage was <0.6 V, corresponding to half of the band gap width of silicon (1.12 eV). Afterward, currents of different devices increased with the voltage, and the currents of In- and Ga-doped devices were significantly higher than that of the original device. When the voltage reaches 1.5 V, the Si–In/SiO_2_/Si device achieved a current value of 0.23 nA, which is about 2.5 times the current of the original device. Meanwhile, the current of the Ga-doped device was 0.29 nA. To confirm the effectiveness of the In/Ga dopants in this work, we compared the calculated data in I-V curves of different modified devices in other works, as shown in Table 1. The In/Ga-modified devices have certain advantages. The I-V curve results theoretically confirmed that In and Ga dopants positively impacted the power density of SABs.

## 3. Method

The DFT analysis was conducted using the Atomistix Toolkit (ATK) code, and the generalized gradient approximation (GGA) was performed using Perdew–Burke–Ernzerhof (PBE) [34,35,36,37,38,39]. The k-point grid was tested following the procedure of previous work [24,25]. The constructed Si/SiO_2_ and doped Si/SiO_2_ models adopted the 4 × 4 × 1 k-point grid. The cut-off energy of 125 Ha was adopted for the pseudopotentials of Si, In, Ga, and O with an energy tolerance of 0.002 eV. The constructed Si (100) substrate had 28 atomic layers comprising 112 Si atoms with a vacuum layer thickness of 20 Å and lattice constants a = b = 7.68 Å. All atoms were fully relaxed in the model structure with a force tolerance of 0.02 eV/Å [24,25,39]. The Si electrode was first optimized, and the number of Si layers and the thickness of the vacuum layer were further adjusted by surface cleaving. The projected density of states is a way to visualize the contribution of different orbits to the density of states, which is defined as $DM\left(\varepsilon \right)=\sum_{n}\delta \left(\varepsilon -\varepsilon n\right)\langle \psi n\left|\overset{\wedge }{P}\right|\psi n\rangle$. The density of states can be obtained using the projection operator $\overset{\wedge }{P}M$ on the subspace M as $D\left(\varepsilon \right)=\sum_{n}\delta \left(\varepsilon -\varepsilon n\right)$ [34,35,36,37,38,39]. The projected local density of states (PLDOS) and I-V curves were calculated following the method of previous works [24,25]. For the device model construction, various Si/SiO_2_ interfaces were constructed using the Green function surface model. PLDOS was calculated using the electronic structure of electrode surface boundary matching, and the I-V curves were calculated based on different Si/SiO_2_/Si devices with bias voltage settings completed. I-V curves were obtained by calculating the current at different bias voltages using different device models. In QuantumATK, the bias points can be directly sampled. The voltage bias was 0–1.5 V with 16 points [24,25].

## 4. Conclusions

In this work, we performed DFT calculations on various established Si–In/SiO_2_ and Si–Ga/SiO_2_ models to evaluate the influence of In and Ga dopants on the performance of SABs. The adsorption energy of the doped silicon electrode was higher than that of the pure silicon electrode on the passivation layer, indicating that the In and Ga dopants would suppress the surface passivation. Moreover, the band gap width of the SiO_2_ layer in the In/Ga-doped interface was significantly narrower than that of the original interface. In and Ga introduced new energy levels in the SiO_2_ interface, facilitating electrons migration through the anode interface. The adsorption energy of the H_2_O molecule at the doped Si substrate significantly increased, indicating that the modification inhibited self-corrosion and the actual energy density of Si–air batteries. In addition, the I-V curve of various devices confirmed that In and Ga dopants significantly increased the current through the interface and improved the power density of SABs. Hence, Si anodes modified by dopants, such as In and Ga, protect SABs and provide guidelines for rationally designing Si-based composite anodes in air batteries.

At present, there is still relatively little theoretical research on anode doping in silicon–air batteries, and the working mechanism of doped silicon-based composite anodes at the atomic level remains unclear. This work provides a new approach to anode doping. In addition, we plan to prepare composite anodes containing indium and gallium and assemble them into silicon–air batteries to test the theoretical results.

## Figures and Tables

**Table 1:** I-V curve data of devices compared with other works.

Modification Strategy	Current	Bias Voltage	References
Si/SiO_2_/Si	0.086 nA	1.5 V	/
Si-In/SiO_2_/Si	0.23 nA	1.5 V	
Si-Ga/SiO_2_/Si	0.29 nA	1.5 V	
Si-Zn/SiO_2_/Si	0.43 nA	1.5 V	[24]
Si-Fe/SiO_2_/Si	0.20 nA	1.5 V	
Si/Ge-2/SiO_2_/Si	0.19 nA	1.5 V	[25]
Si/Ge-4/SiO_2_/Si	0.19 nA	1.5 V	
Si/Ge-12/SiO_2_/Si	0.20 nA	1.5 V	

## Data Availability

Not applicable.
